# Correspondence: Variations in ocean heat uptake during the surface warming hiatus

**DOI:** 10.1038/ncomms12541

**Published:** 2016-08-19

**Authors:** Xianyao Chen, Ka-Kit Tung

**Affiliations:** 1 Key Laboratory of Physical Oceanography, Ocean University of China, Qingdao 266100, China; 2 Department of Applied Mathematics, University of Washington, Seattle, Washington 98195, USA

Nature Communications 7:12541 doi: 10.1038/ncomms12541 (2016); Published 08 19 2016

Liu *et al*.^1^ re-examined the variation of ocean heat uptake during the global warming hiatus period or 1998–2012. They used the same observational data set (Ishii *et al*.^2^) and analysed the ocean heat uptake the same way as the earlier work of Chen and Tung^3^, but reached an opposite conclusion. We discuss here why we do not agree with their interpretation.

The result in their Fig. 2 is almost the same as in Fig. 1 of our previous publication. As shown here in Fig. 1 for the variation of the 300–1,500 m ocean heat content (OHC), they are almost identical. The only difference is the subtraction of a different climatology, which has the effect of offsetting their curves relative to ours. The offset does not matter as far as the linear trends that they focused on are concerned.

Table 1 here shows the linear trends of OHC change in the 300–1,500 m layer for the period of rapid surface global warming 1970–1997 and hiatus period 1998–2012, as well as those for the two periods combined 1970–2012. The periods were chosen by Liu *et al*.^1^. During the period of rapid surface warming (1970–1997), there was much smaller global ocean heat uptake: 0.12 in units of 10^23^ J per decade, compared with the uptake of 0.58 in the same units for the period of surface hiatus that followed. The latter period has more than four times as large an OHC increase globally. The Pacific Ocean has a very small actual OHC increase in either period: 0.06 and 0.05, respectively. However, when expressed in percentage form, one can claim that the Pacific was responsible for 50% of the global heat uptake during the first period.

In contrast, the heat uptake in the Atlantic into the 300–1,500 m layer increased dramatically, by a factor of almost 4, during the surface hiatus period as compared with the rapid surface warming period. Although the Southern Ocean data were less trustworthy, the Ishii data used by them showed even more dramatic increase in the heat uptake during the second period. Chen and Tung^3^ pointed out that it was these variations of the vertical distribution of OHC that account for the different surface warming behaviours between a period of rapid surface warming and a period of hiatus. In comparison, the Pacific and Indian Oceans are seen to play comparatively minor roles in the change in OHC in the intermediate layers (300–1,500 m).

The arguments of Liu *et al*.^1^ were based on percentages instead of the actual magnitudes of change in OHC and, as a result, masked the change in behaviour between the two periods. The change was further obscured when the authors combined the two periods together. For the layer 300–1,500 m, they found that the percentage of the linear warming trend of the OHC in the Atlantic, Southern Ocean, Pacific and Indian Ocean to the global OHC warming trend during 1998–2012 are 33.8, 40.7, 8.2 and 15.8%, respectively, which were deemed similar to those during the longer period from 1970 to 2012—30.7, 41.3, 13.5 and 5.4%, respectively. Although they agreed with Chen and Tung^3^ on the larger role played by the Atlantic and Southern Oceans, they argued that the larger heat uptakes in these ocean basins have not changed between the two periods and that fact should justify the interpretation that ‘the deep heat penetration in these two basins is not unique to the hiatus but is characteristic of anthropogenic warming'. They neglected to mention that in terms of actual OHC magnitudes the hiatus period is clearly distinct from the prior decades. The fact that their argument is false can be demonstrated in a hypothetical case where one ocean basin, say the Atlantic, does all the heat uptake observed for the globe during the two periods in the 300–1,500 m layer. Thus, this Atlantic Ocean's percentage of the global heat uptake, 100%, does not change in the two periods and in the combined period. It is clearly wrong to argue then that the OHC change in this ocean, which is 0.58 during the hiatus period, is ‘not unique to the hiatus', when compared with the much smaller heat uptake of 0.12 in the earlier non-hiatus period.

In addition to the results based on Ishii data, Liu *et al*.^1^ presented modelling results based on Community Earth System Model simulations. That model, such as the Community Climate System Model 4 analysed by Chen and Tung^3^, has a much diminished multidecadal internal variability in the Atlantic Ocean's 300–1,500 m OHC when compared with the observation. This can also be seen in their Fig. 2 (2d versus 2e): the observed Atlantic OHC change during the hiatus period is much larger than the modelled hiatus group, by a factor of 6 during 1985–2005, when the Atlantic Meridional Overturning Circulation (AMOC) subducted a large amount of heat as it sped up^3^,^4^. Next, as AMOC slowed after 2005 (ref. 5), the Southern Ocean picked up the heat subduction, by a factor of 2–3 more than the model (Fig. 2g versus 2h). (The Argo floats reached adequate coverage even in the Southern Ocean after 2005). The model is missing these variations in heat uptake corresponding to the observed variation in AMOC, most probably an internal variability. The perception of the AMOC as an unchanging mechanism by Liu *et al*.^1^ runs counter to the *in situ* and altimetry observations of AMOC variations. When the internal variability is weak, forced response plays a bigger role in ocean heat uptake in the model. One should not have concluded based on the model four-member ensemble mean with reduced internal variability that the observed variation in OHC is forced.

The positive radiative imbalance at the top of the atmosphere, ∼0.5 Wm^−2^ (refs 6, 7, 8), is mostly driven by the increases in anthropogenic greenhouse gases reducing the infrared emission to the space. This imbalance resides mostly in the oceans, as the heat capacity of the atmosphere, land and cryosphere is small^9^. Energy balance relates the top of the atmosphere imbalance to the time rate of change of total OHC. (It is noteworthy that the top of the atmosphere values measured by satellites come with very large error bars, even larger than the ones associated with the observed OHC changes from various ocean data sets. Thus, for practical purposes the latter are often used to estimate the former^10^) The fact that the total OHC, as approximated by the 0–1,500 m OHC, is increasing steadily since 1970 is to be expected, given the consensus of a positive radiative imbalance. This feature in the Fig. 2 of Liu *et al*.^1^ was previously shown in Fig. 1 of Chen and Tung^3^ using the same data set and discussed in terms of the energy budget of the earth, and in Balmaseda *et al*.^11^ using reanalysis data. What was of interest with regards to answering the question of what caused the period of surface warming slowdown is the variation in the vertical distribution of the OHC. During the surface hiatus period, 1999–2012, it was found by Chen and Tung^3^ that the vertical distribution of the OHC was such that more heat was sequestered in the intermediate layers of the global oceans and less near the upper 300 m layer and the surface. During the prior decades, less was sequestered and hence more heat remained to warm the surface and the upper layer of the oceans. The ocean basins with the largest changes in heat content in the 300–1,500 m layer are the Atlantic and the Southern Ocean. It is meaningless to dismiss this change in behaviour as simply anthropogenic warming, as almost all such heat changes in recent multidecadal time scales was anthropogenic in origin, with the exception of the smaller solar forcing changes and shorter-term events such as volcanic eruptions and El Niño-Southern Oscillation variations.

Table 1 here and Fig. 1 of Chen and Tung^3^ show that there were large variations in the vertical distribution of heat between the hiatus and non-hiatus periods in the observations, much more than in the model results presented. The presentation by Liu *et al*.^1^ obscured such a variation in observations by comparing the hiatus period with a longer period that includes the hiatus period, and by using percentages instead of actual magnitudes of OHC. The actual magnitudes should have been used when tracking where the ‘missing' heat^12^ went.

## Data availability

The Ishii data is publicly available at http://rda.ucar.edu/datasets/ds285.3/.

## Additional information

**How to cite this article:** Chen, X. Y. & Tung, K.-K. Correspondence: Variations in ocean heat uptake during the surface warming hiatus. *Nat. Commun.* 7:12541 doi: 10.1038/ncomms12541 (2016).

## Figures and Tables

**Figure 1:**
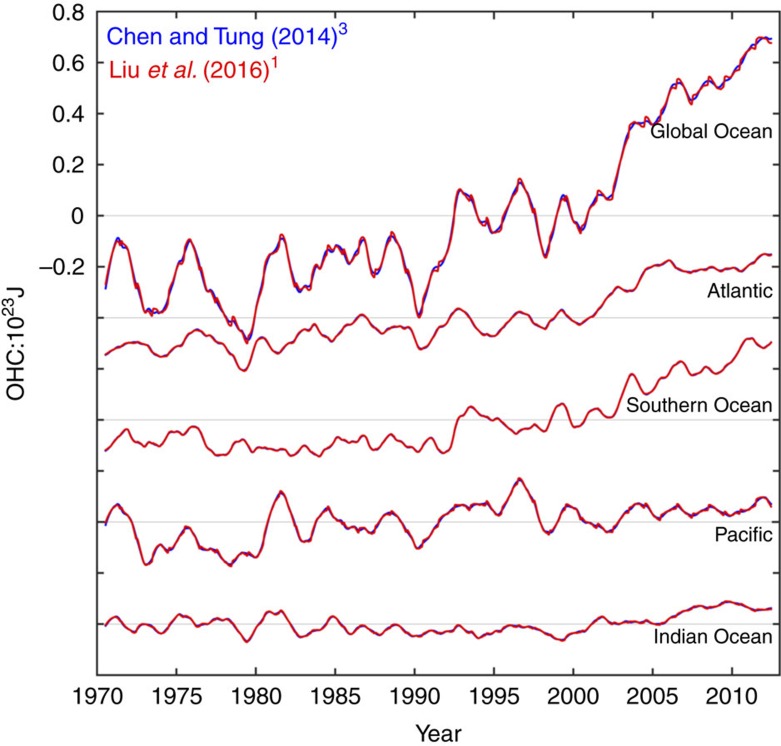
Comparing ocean heat content. Comparison of ocean heat content (OHC) change in the 300–1,500 m layer in the global ocean, Atlantic, the Southern Ocean, Pacific and the Indian Ocean, from Chen and Tung^3^ (in blue), and Liu *et al*.^1^ (in red). As Chen and Tung^3^ subtracted a warmer climatology based on the monthly mean of 1970–2012, as compared with the 1970 values used by Liu *et al*.^1^, the curves from Liu *et al*.^1^ are shifted up, for ease of comparison.

**Table 1:** Linear trend of OHC change in the 300–1,500 m layer in global and regional oceans in the unit of 10^23^ J per decade.

	**1970–1997**	**1998–2012**	**1970–2012**
Global Ocean	0.12	0.58	0.22
Atlantic	0.05	0.19	0.08
Southern Ocean	0.02	0.23	0.09
Pacific	0.06	0.05	0.03
Indian Ocean	0.01	0.09	0.01

OHC, Ocean Heat Content.
